# Secondhand smoke exposure is associated with the risk of hypertensive disorders of pregnancy: the Japan Environment and Children’s Study

**DOI:** 10.1038/s41440-022-01144-3

**Published:** 2023-02-03

**Authors:** Kosuke Tanaka, Hidekazu Nishigori, Zen Watanabe, Kaoh Tanoue, Noriyuki Iwama, Michihiro Satoh, Takahisa Murakami, Tetsuro Hoshiai, Masatoshi Saito, Satoshi Mizuno, Kasumi Sakurai, Mami Ishikuro, Taku Obara, Nozomi Tatsuta, Ikuma Fujiwara, Shinichi Kuriyama, Takahiro Arima, Kunihiko Nakai, Nobuo Yaegashi, Hirohito Metoki, and the Japan Environment and Children’s Study Group

**Affiliations:** 1 Department of Obstetrics and Gynecology, Tohoku University Graduate School of Medicine, Sendai, Miyagi Japan; 2 Environment and Genome Research Center, Tohoku University Graduate School of Medicine, Sendai, Miyagi Japan; 3 Fukushima Medical Center for Children and Women, Fukushima Medical University, Fukushima, Japan; 4 Division of Public Health, Hygiene and Epidemiology, Faculty of Medicine, Tohoku Medical and Pharmaceutical University, Sendai, Miyagi Japan; 5 Tohoku Medical Megabank Organization, Tohoku University, Sendai, Miyagi Japan; 6 Department of Pharmaceutical Sciences, Tohoku University Hospital, Sendai, Miyagi Japan; 7 International Research Institute of Disaster Science, Tohoku University, Sendai, Miyagi Japan

**Keywords:** Hypertension, Hypertensive disorders of pregnancy, Japan Environment and Children’s Study, Secondhand smoke smoking

## Abstract

Hypertensive disorders of pregnancy (HDP) are associated with poor maternal and neonatal prognoses. Although several studies have indicated an effect of secondhand smoke (SHS) exposure on HDP, such evidence is lacking in Japan. Therefore, we analyzed data from the Japan Environment and Children’s Study, a large-scale epidemiological investigation, to elucidate a possible link between SHS exposure and HDP risk. Data were obtained from the all-birth fixed datasets and included information on 104,062 fetuses and their parents. SHS exposure was assessed in terms of the frequency (rarely, 1–3, or 4–7 days/week) and the daily duration of exposure (<1, 1–2, or ≥2 h(s)/day). Modified Poisson regression model analyses were performed with adjustment for known risk factors for HDP. Additionally, the population attributable fractions (PAFs) of SHS exposure and maternal smoking to HDP prevalence were estimated. The relative risks of developing HDP among individuals with SHS exposures of 4–7 days/week and ≥2 h/day were 1.18 and 1.27 (95% confidence interval: 1.02–1.36 and 0.96–1.67), respectively, compared to the reference groups (rare exposure and <1 h/day). The PAFs for the risk of HDP due to SHS exposure and perinatal smoking were 3.8% and 1.8%, respectively. Japanese women with greater exposure to SHS have a higher risk of HDP after adjustment for possible confounding factors; thus, relevant measures are required to reduce SHS exposure to alleviate HDP risk.

**Figure:**
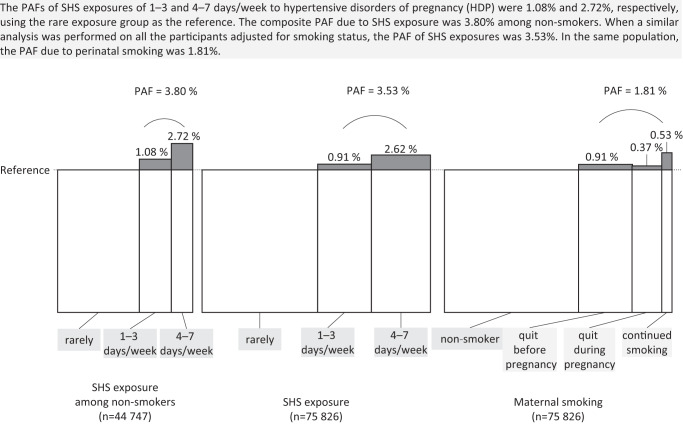
The association between second-hand smoking exposure and hypertensive disorders of pregnancy risk was analyzed using the JECS data. The relative risks in 4–7 days/week and ≥2 h/day of SHS exposures were 1.18 and 1.27, respectively. The PAFs due to SHS exposure and maternal smoking were 3.80% and 1.81%, respectively.

The association between second-hand smoking exposure and hypertensive disorders of pregnancy risk was analyzed using the JECS data. The relative risks in 4–7 days/week and ≥2 h/day of SHS exposures were 1.18 and 1.27, respectively. The PAFs due to SHS exposure and maternal smoking were 3.80% and 1.81%, respectively.

## Abstract (web summary)

The association between second-hand smoking exposure and hypertensive disorders of pregnancy risk was analyzed using the JECS data. The relative risks in 4–7 days/week and ≥2 h/day of SHS exposures were 1.18 and 1.27, respectively. The PAFs due to SHS exposure and maternal smoking were 3.80% and 1.81%, respectively.

## Introduction

Hypertensive disorders of pregnancy (HDP) are observed in ~5–10% of pregnant women and are associated with a poor maternal and neonatal prognosis due to premature delivery, stillbirth, impaired fetal growth, and maternal death [1]. HDPs are also associated with specific risk factors, including first pregnancy; higher body mass index (BMI) or age; and preexisting dyslipidemia, diabetes mellitus, or renal disease; [2, 3] and socioeconomic status [4]. Although the cause of HDP remains unknown, current hypotheses postulate a placental pathogenesis. Abnormal placentation leading to preeclampsia is marked by the failure of trophoblasts to induce physiologic dilatation and remodeling of the spiral arteries, resulting in reduced placental blood flow [5].

Smoking during pregnancy is one of the most important risk factors for various adverse birth outcomes, including low birth weight and preterm birth [6]. Furthermore, several studies have indicated an influence of secondhand smoke (SHS) exposure during pregnancy on birth outcomes. For example, Polanska et al. reported that SHS exposure during pregnancy has a negative impact on child psychomotor development within the first two years of life [7]. Additionally, Windham et al. reported that high SHS exposure (more than 7 h/day) is associated with preterm birth [8]. Studies regarding the effect of SHS exposure on HDP risk were conducted in Norway [9] and North America [10] with inconsistent results. Studies on the influence of SHS exposure on HDP risk in Japan are lacking. A recent meta-analysis in Japan revealed that smoking is a risk factor for HDP [11]. We believe it is necessary to examine the effects of secondhand smoke on HDP using Japanese data.

The Japanese Ministry of the Environment launched the Japan Environment and Children’s Study (JECS), an ongoing large-scale epidemiological investigation, in January 2011 [12]. The JECS invited ~100,000 pregnant women and their partners to participate over a period of 3 years following collection of biological samples; data on their children were subsequently obtained until the age of 13 years. Herein, we analyzed JECS data to elucidate a possible link between SHS exposure and HDP risk.

Point of view

- **Clinical relevance**
  The PAFs for the risk of HDP from SHS and perinatal smoking were 3.8% and 1.8%, respectively, with SHS having a greater impact.
- **Future direction**
  Relevant strategies are needed to reduce SHS exposure among women of reproductive age.
- **Consideration for the Asian population**
  As the effects of SHS may be more pronounced in Asians, appropriate strategies need to be designed.

## Methods

### Study design

This study was part of the JECS, an ongoing nationwide birth cohort study. The JECS was approved by the Ministry of the Environment’s Institutional Review Board on Epidemiological Studies on April 6, 2010 (no. 100406001), and the ethics committees of all participating institutions. Written informed consent was obtained from all participants. Participants were recruited from January 2011 to March 2014 at 15 regional centers located in Hokkaido, Miyagi, Fukushima, Chiba, Kanagawa, Koshin, Toyama, Aichi, Kyoto, Osaka, Hyogo, Tottori, Kochi, Fukuoka, and South Kyushu and Okinawa. The analysis is based on the jecs-ta-20190930 datasets, including information on 104,062 fetuses and their parents. The baseline profiles of participants were described in a previous study [13].

### Data collection

We obtained pregnancy-related information, including the weight before pregnancy, from two self-report questionnaires (MT1 and MT2). The MT1 questionnaire was completed on enrollment in the JECS, while the MT2 questionnaire comprised data pertaining to the second and third trimester. The mean (standard deviation) gestational age at the time of completion of the MT1 and MT2 questionnaires was 16.4 (8.0) and 27.9 (6.5) weeks, respectively. Data from the Dr0m questionnaire were obtained from medical records after delivery via the cooperation of health care providers or research coordinators from regional centers. The Dr0m questionnaire was designed to gather data on the outcomes of the pregnancy and offspring, including the maternal age and weight at delivery, complications during pregnancy, birth weight of child, and length of gestation. This information was transcribed by instructed physicians, midwives/nurses, and/or research coordinators from medical records. Incomplete questionnaires were completed via a face-to-face or telephone interview.

We obtained the following data from the MT1 questionnaire: parity, BMI (kg/m^2^), smoking and drinking habits of the participants and their partners, frequency and duration of SHS exposure, educational levels of the participants and their partners, Kessler psychological distress scale (K6) score, and complications before pregnancy (i.e., hypertension, renal diseases, dyslipidemia, and diabetes mellitus). Sodium (mg/day), potassium (mg/day), calcium (mg/day), magnesium (mg/day), salt (g/day), and total energy (kJ/day) intake were obtained from the food frequency questionnaire (FFQ) on the MT1 questionnaire. The association between nutrient intake and HDP based on the FFQ has been reported previously [14, 15]. Socioeconomic status data, such as family income and the educational level of the participants and their partners, were obtained from the MT2 questionnaire. The K6 is a widely used screening scale for psychological distress in the general population [16]. The Japanese version of the K6 was recently developed using the standard back-translation method [17]. Details regarding K6 have been previously described [18].

### Participants

**Fig. 1:**
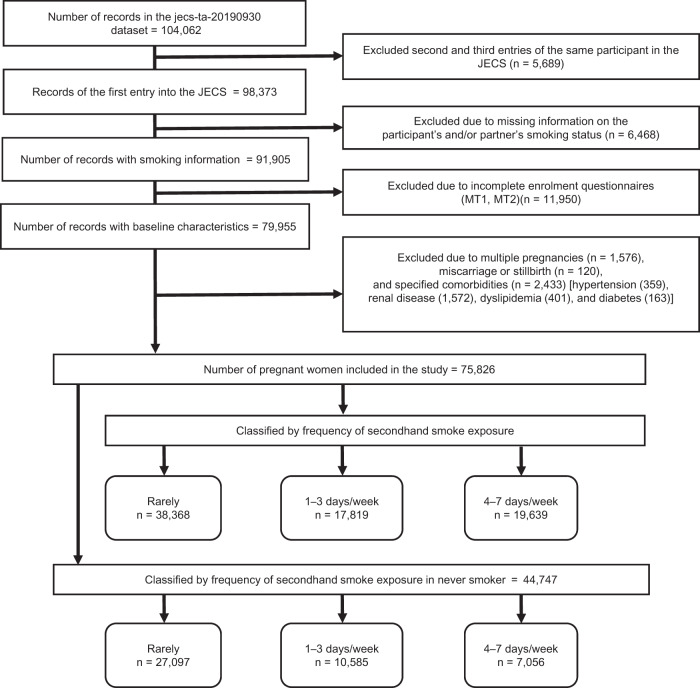
The participant selection flowchart. The final dataset comprised primary data from 75,826 pregnant women included in the Japanese Environment and Children’s Study (JECS)

We excluded women who participated in the JECS twice (*n* = 5689), those with missing data regarding their smoking status (active smoking of participants and partners, maternal SHS exposure) (*n* = 6468) and those with missing baseline data (Table 1 variables) (*n* = 11,950). We focused on low-risk pregnancies; thus, we excluded women with multiple pregnancies (*n* = 1576) and those with a history of miscarriage or stillbirth (*n* = 120). Finally, we excluded women with specified comorbidities (*n* = 2433), including diagnosed hypertension (*n* = 359), renal diseases (*n* = 1572), diabetes mellitus (*n* = 163), and dyslipidemia (*n* = 401), with some overlap among the comorbidities. The records of the remaining 75,826 women were analyzed. Excluding 31,079 smokers, the main analysis included 44,747 subjects. Subanalyses were also performed for the 75,826 individuals, including smokers. Figure 1 shows the participant selection flowchart.

**Table 1:** Basic characteristics (total *n* = 44,747)

		Frequency of SHS exposure before pregnancy		
		rarely	1–3 days/week	4–7 days/week
		(*n* = 27,097)	(*n* = 10,585)	(*n* = 7065)
Duration of SHS exposure (/day)(%)				
	<1 h	99.91	84.38	71.86
	1–2 h	0.06	11.78	15.92
	≥2 h	0.03	3.84	12.22
Participant’s background				
Age (years)				
	mean (SD)	32.15 (4.55)	30.99 (4.83)	30.28 (5.36)
	<20	0.17	0.44	1.76
	20–24	4.24	8.27	12.92
	25–29	24.93	31.32	30.62
	30–34	39.20	35.22	31.82
	35–39	26.15	20.69	18.70
	≥40	5.31	4.06	4.19
Primipara (%)		39.90	50.63	53.76
BMI before pregnancy (kg/m^2^) (%)				
	mean (SD)	20.91 (2.92)	21.17 (3.16)	21.34 (3.37)
	<18.5	16.75	15.95	15.07
	18.5–24.9	75.15	74.13	73.55
	≥25.0	8.10	9.92	11.38
Weight gain during pregnancy (kg) (SD) (*n* = 43,732)		9.78 (6.38)	9.92 (3.69)	10.17 (3.97)
Alcohol consumption at entry (%)				
	no drinking	42.19	38.45	39.35
	quit before or during pregnancy	47.96	50.77	50.30
	continued drinking	9.85	10.78	10.35
K6 score ≥13 (%)		2.35	2.72	3.77
Educational level (%)				
	junior high school	0.69	1.62	3.35
	high school	19.14	25.73	37.58
	college	80.17	72.66	59.07
Family income (JPY) (%)				
	<200 × 10^4^	2.53	4.48	7.30
	200–399 × 10^4^	27.36	32.10	38.94
	400–599 × 10^4^	35.38	34.08	30.66
	600–799 × 10^4^	20.23	17.38	13.80
	≥800 × 10^4^	14.50	11.96	9.30
Sodium intake (mg/day)	median (inter quartile range)	2836 (2150–3728)	2887 (2158–3864)	2902 (2117–3944)
Pottasium intake (mg/day)	median (inter quartile range)	2131 (1624–2812)	2116 (1590–2826)	2075 (1505–2792)
Calcium intake (mg/day)	median (inter quartile range)	471 (332–656)	459 (321–648)	439 (294–634)
Magnecium intake (mg/day)	median (inter quartile range)	222 (173–287)	221 (171–289)	217 (162–284)
Salt intake (g/day)	median (inter quartile range)	7.1 (5.4–9.8)	7.3 (5.4–9.8)	7.3 (5.3–9.9)
Total Energy intake (kJ/day)	median (inter quartile range)	7029 (5809–8626)	7194 (5847–8860)	7140 (5721–9022)
History of HDP (%)		1.16	1.21	1.05
History of GDM (%)		0.64	0.43	0.57
Patner’s Background				
Partner’s smoking before pregnancy (%)				
	no smoking	43.64	31.11	17.31
	quit before pregnancy	30.36	23.09	13.43
	quit during pregnancy	1.41	2.28	2.36
	continued smoking	24.59	43.52	66.89
Partner’s educational level (%)				
	junior high school	2.03	4.09	8.63
	high school	27.20	33.25	42.25
	college	70.77	62.66	49.12
Outcome				
Prevalence of HDP (%)		2.58	2.96	3.47

*SD* standard deviation, *BMI* body mass index, *SHS* secondhand smoke, *JPY* Japanese yen, *HDP* hypertensive disorders of pregnancy, *K6* Kessler psychological distress scale

### Secondhand smoke exposure

Information on SHS exposure was obtained from the self-administered MT1 questionnaire, which asked the following question: Prior to this pregnancy, how many times per week were you exposed to smoke from cigarettes smoked by others in your home, at work, or indoors when you were away from home? In the questionnaire, SHS exposure was assessed by asking how often the participant was exposed to SHS per week (rarely, 1–3, or 4–7 days/week). Exposed participants were asked to respond to a question regarding the average duration of SHS exposure per day (<1, 1–2, or ≥2 h(s)/day).

### Hypertensive disorders of pregnancy

Information on HDP was obtained from the Dr0m questionnaire. This questionnaire only revealed whether the participant was diagnosed with HDP; data regarding specific HDP were not provided (i.e., gestational hypertension, preeclampsia, or superimposed preeclampsia and eclampsia). In the JECS, HDP was defined as hypertension (blood pressure [BP] ≥ 140/90 mmHg), with or without proteinuria (≥300 mg/24 h) emerging after 20 weeks gestation but resolving up to 12 weeks postpartum, or as eclampsia [19].

### Statistical analysis

Statistical analyses were performed using SAS software (version 9.4, SAS Institute Inc., Cary, NC). Baseline characteristics are reported as percentages, means and standard deviations, or medians and interquartile ranges, as appropriate. Women with rare exposure to SHS were considered to be reference group in analyses of the effect of SHS exposure frequency on HDP risk, and women with SHS exposure of less than 1 h/day were considered to be the reference group in analyses of the effect of SHS exposure duration on HDP risk. We used a modified Poisson regression model to calculate crude relative risks (RR) and adjusted relative risks (aRR) [20]. In model 1, we adjusted for other risk factors for HDP, including age, parity, BMI before pregnancy, family income, maternal educational levels, drinking, smoking status, Na, K, Ca, Mg, total energy intake, and K6 score at entry. In model 2, we adjusted for a history of HDP and gestational diabetes mellitus (GDM) in addition to the factors in model 1. In model 3, the partner’s educational level was adjusted.

Additionally, we estimated the population attributable fractions (PAFs) and the 95% confidence intervals (CIs) of SHS exposure and maternal smoking to HDP prevalence using the NLEST macro. Using the aRR from model 3, the PAF was calculated as follows:

$$Estimated\;excess\;HDP\;case = pc \times \left( {aRR - 1} \right)/aRR$$

$$PAF = estimated\;excess\;HDP\;case/all\;HDP\;cases \times 100$$

$Composite\;PAF = sum\;of\;estimated\;excess\;HDP\;cases\;of\;each\;category/allHDP\;cases \times 100$(where pc is the proportion of HDP cases arising from each category)

## Results

Table 1 shows the basic characteristics of the study participants. The distribution of SHS exposure frequency was as follows: rarely, 27,097 (60.6%); 1–3 days/week, 10,585 (23.7%); and 4–7 days/week, 7065 (15.8%). The number of primiparous women was the lowest in the rare exposure group, while those with frequent SHS exposure had less education and income. All baseline measurements were significantly different among the three groups (all *p* < 0.05). The prevalence of women with HDP according to SHS exposure frequency was significantly different among the three groups as follows: rarely, 2.6%; 1–3 days/week, 3.0%; and 4–7 days/week, 3.5% (*p* < 0.01). Supplementary Table 1 presents the results for all 75,826 participants, regardless of maternal smoking status.

Table 2 and Supplementary Table 2 show the univariate analyses on HDP risk in nonsmokers and all participants, respectively. Compared to women with rare SHS exposure, pregnant women with SHS exposure of 1–3 and 4–7 days/week showed a higher risk of developing HDP (RR: 1.15, 95% confidence interval [CI]: 1.01–1.31 and RR: 1.35, 95% CI: 1.17–1.55, respectively). Additionally, pregnant women with SHS exposure of 1–2 or ≥2 h/day had a significantly higher risk of developing HDP (RR: 1.49, 95% CI: 1.21–1.83 and RR: 1.48, 95% CI: 1.13–1.95, respectively) than those with SHS exposure of <1 h/day.

**Table 2:** Results of the univariate analyses on HDP for the covariates (total *n* = 44,747)

		RR	95% CI
Frequency of SHS exposure	rarely	Reference	
	1–3 days/week	1.148	1.007–1.309
	4–7 days/week	1.346	1.167–1.554
Duration of SHS exposure	<1 h	Reference	
	1–2 h	1.489	1.213–1.826
	≥2 h	1.481	1.125–1.950
Age (years)	<20	Reference	
	20–24	0.975	0.397–2.395
	25–29	0.976	0.407–2.338
	30–34	1.054	0.441–2.520
	35–39	1.597	0.668–3.820
	≥40	2.345	0.969–5.678
Parity	primipara	1.953	1.746–2.184
	multipara	Reference	
BMI (kg/m^2^)	<18.5	0.636	0.524–0.771
	18.5–24.9	Reference	
	≥25.0	3.000	2.642–3.407
Alcohol consumption	no drinking	Reference	
	quit before or during pregnancy	0.912	0.813–1.023
	continued drinking	0.882	0.726–1.073
K6 score	<13	Reference	
	≥13	1.080	0.779–1.497
Maternal educational level	junior high school	1.053	0.656–1.690
	high school	1.135	1.002–1.286
	college	Reference	
Family income (×10^4^ JPY)	<200	Reference	
	200–399	0.949	0.776–1.160
	400–599	0.855	0.699–1.045
	600–799	0.995	0.803–1.233
	≥800	0.873	0.670–1.138
Sodium intake	Q1	Reference	
	Q2	0.865	0.729–1.027
	Q3	0.850	0.716–1.011
	Q4	0.898	0.758–1.064
	Q5	0.990	0.839–1.168
Pottasium intake (mg/day)	Q1	Reference	
	Q2	0.941	0.791–1.119
	Q3	1.030	0.869–1.220
	Q4	0.972	0.818–1.154
	Q5	0.984	0.829–1.168
Calcium intake (mg/day)	Q1	Reference	
	Q2	1.180	0.999–1.394
	Q3	1.014	0.853–1.206
	Q4	0.945	0.793–1.127
	Q5	0.924	0.774–1.103
Magnesium intake (mg/day)	Q1	Reference	
	Q2	0.999	0.840–1.061
	Q3	0.989	0.831–1.118
	Q4	1.067	0.900–1.037
	Q5	0.997	0.000–1.087
Salt intake (g/day)	Q1	Reference	
	Q2	0.845	0.710–1.006
	Q3	0.872	0.735–1.034
	Q4	0.891	0.753–1.054
	Q5	0.991	0.840–1.169
Total Energy intake (kJ/day)	Q1	Reference	
	Q2	0.912	0.764–1.088
	Q3	1.019	0.858–1.211
	Q4	0.968	0.813–1.152
	Q5	1.124	0.950–1.329
History of gestational diabetes		1.657	0.951–2.170
History of HDP		6.762	5.571–8.207
Partner smoking status	no smoking	Reference	
	quit before pregnancy	0.962	0.835–1.109
	quit during pregnancy	0.906	0.582–1.410
	continued smoking	1.048	0.923–1.191
Partner’s educational level	junior high school	1.210	0.916–1.600
	high school	1.171	1.043–1.315
	college	Reference	

*RR* relative risk, *CI* confidence interval, *HDP* hypertensive disorders of pregnancy, *JPY* Japanese yen, *K6* Kessler psychological distress scale, *SHS* secondhand smoke, *BMI* body mass index

Table 3 and Supplementary Table 3 show that the relative risk for SHS exposure and HDP risk changed as a result of adjusting for possible confounding factors. In the multivariate analyses regarding the impact of SHS exposure frequency on HDP risk, after adjusting for maternal confounding factors (model 1), a previous history of GDM and HDP (model 2), and partner factors (model 3), pregnant women with SHS exposure of 4–7 days/week had a higher risk of developing HDP (aRR: 1.20, 95% CI: 1.03–1.39; aRR: 1.19, 95% CI: 1.03–1.38; aRR: 1.18, 95% CI: 1.02–1.36, respectively) than the reference group. A linear trend was observed between SHS exposure frequency and HDP risk after adjustment for all models (*p* < 0.05).

**Table 3:** Association between the frequency and duration of secondhand smoke exposure and HDP among nonsmokers

		Relative Risk	95% CI
	Frequency of maternal SHS exposure before pregnancy		
Crude	rarely	Reference	
	1–3 days/week	1.148	1.007–1.309
	4–7 days/week	1.346	1.167–1.554
Model 1	rarely	Reference	
	1–3 days/week	1.076	0.943–1.228
	4–7 days/week	1.197	1.032–1.387
Model 2	rarely	Reference	
	1–3 days/week	1.052	0.921–1.200
	4–7 days/week	1.189	1.027–1.376
Model 3	rarely	Reference	
	1–3 days/week	1.046	0.917–1.195
	4–7 days/week	1.177	1.017–1.363
	Duration of maternal SHS exposure before pregnancy		
Crude	<1 h	Reference	
	1–2 h	1.489	1.213–1.826
	≥2 h	1.481	1.125–1.950
Model 1	<1 h	Reference	
	1–2 h	1.315	1.073–1.613
	≥2 h	1.304	0.992–1.714
Model 2	<1 h	Reference	
	1–2 h	1.282	1.049–1.566
	≥3 h	1.276	0.968–1.681
Model 3	<1 h	Reference	
	1–2 h	1.270	1.039–1.551
	≥2 h	1.266	0.960–1.670

Model 1: Adjusted for age, parity, body mass index before pregnancy, family income, maternal educational level, drinking status, Na, K, Ca, Mg, and total energy intake, and K6 score

Model 2: Adjusted for a history of GDM and HDP in addition to the factors in Model 1

Model 3: Adjusted for partner’s educational level in addition to the factors in Model 2

*SHS* secondhand smoke, *HDP* hypertensive disorders of pregnancy, *GDM* gestational diabetes mellitus, *K6* Kessler psychological distress scale, *CI* confidence interval

In the multivariate analyses regarding the impact of SHS exposure duration on HDP risk, with adjustment as per Models 1, 2, and 3, the estimated risk of HDP development among pregnant women with SHS exposure of ≥2 h/day was ~1.3 times higher than that of the reference group (aRR: 1.30, 95% CI: 0.99–1.71; aRR: 1.28, 95% CI: 0.97–1.68; aRR: 1.27, 95% CI: 0.96–1.67, respectively); this effect was not statistically significant. A linear trend was also observed between the duration of SHS exposure per day and the risk of developing HDP after adjustment for all models (*p* < 0.05).

Supplementary Table 4 shows the results of the association between the frequency of SHS exposure and HDP based on stratification by maternal smoking status with the same adjustment as that in Model 4 (Table 3). No significant association was found between SHS exposure and HDP in any smoking group.

Table 4 shows the PAFs and their 95% CI of SHS exposure and maternal smoking to HDP prevalence. The PAFs of SHS exposures of 1–3 and 4–7 days/week to HDP were 1.1% and 2.7%, respectively (using the rare exposure group as the reference). When a similar analysis was performed on all the participants adjusted for smoking status, the PAFs of SHS exposures of 1–3 and 4–7 days/week to HDP were 0.9% and 2.6%, respectively. In the same population, the PAFs of quitting before pregnancy, quitting during pregnancy, and continued smoking were 0.9%, 0.4%, and 0.5%, respectively (using the never-smoker group as the reference). Among nonsmokers, the composite PAF due to SHS exposure was 3.8%, which was similar (3.5%) to that obtained in the whole population using the same analysis. The PAF due to perinatal smoking was 1.8%.

**Table 4:** PAFs of SHS exposure and maternal smoking to HDP

	*n*	HDP	aRR	95% CI	PAF(%)	95% CI		
SHS exposure in non smokers	44747	1256						
rarely	27097	698	Reference					
1–3 days/week	10585	313	1.046	0.917–1.195	1.083	−2.125–4.291		3.80^d^
4–7 days/week	7065	245	1.177	1.017–1.363	2.721	0.142–5.301		
SHS exposure	75826	2214						
rarely	38368	1013	Reference					
1–3 days/week	17819	537	1.039^b^	0.937–1.153	0.912	−1.577–3.401		3.53^d^
4–7 days/week	19639	664	1.104^b^	0.990–1.230	2.617	-0.327–5.561		
maternal smoking	75826	2214						
non smoker	44747	1256	Reference					
quit before pregnancy	17880	527	1.039^c^	0.9376–1.151	0.907	−1.558 3.372		1.81^d^
quit during pregnancy	9813	316	1.029^c^	0.9016–1.174	0.369	−1.373 2.111		
continued smoking	3386	115	1.120^c^	0.9125–1.374	0.531	−0.481 1.543		

*PAF* population attributable fraction, *HDP* hypertensive disorders of pregnancy, *SHS* secondhand smoke, *GDM* gestational diabetes mellitus, *K6* Kessler psychological distress scale, *aRR* adjusted relative risk, *CI* confidence interval

^a^Adjusted for age, parity, body mass index before pregnancy, family income, educational level of the participant and partner, drinking status of the participant, a history of HDP and GDM, and K6 score

^b^Adjusted for smoking status of the participant in additional to ^a^

^c^Adjusted for SHS exposure (frequency) in additional to ^a^

^d^Composite PAF of each category

Similar results were found when including women with a history of miscarriage, abortion, and diagnosed complications (hypertension, renal diseases, diabetes mellitus, and dyslipidemia) (data not shown).

## Discussion

This study is the first to report an association between SHS exposure and HDP risk in Japan. Women with SHS exposure of 4–7 days/week had a significantly higher risk of developing HDP. Although it was not statistically significant, a linear trend between the duration of SHS exposure per day and the risk of developing HDP after adjustment for possible confounding factors was observed in this nationwide prospective cohort study.

Previous investigations regarding the effects of SHS exposure on HDP risk during pregnancy have shown inconsistent results. For example, Engel et al. reported insufficient evidence regarding the association between SHS exposure and HDP risk; however, SHS exposure data were obtained using a yes/no questionnaire; thus, the quantity and frequency of SHS exposure were not accessible [9]. In contrast, Luo et al. reported that previous smoking and SHS exposure (defined according to the plasma cotinine level) may increase the risk of preeclampsia [10]. Although the utilized cutoff for plasma cotinine was reasonable [21], the effects of previous smoking and SHS exposure could not be estimated separately. Thus, the present study clarifies the association between SHS exposure and HDP risk.

Socioeconomic status is an important confounding factor when considering SHS. In Japan, educational inequalities among current and heavy smokers are more apparent and larger in the younger population than in older generations [22]. Using data from the T-CHILD study, which was conducted in Japan, Jwa et al. found an association between educational levels and BP levels at early gestation, which was mediated by prepregnancy BMI [23]. In the Generation R study, maternal educational levels were significantly associated with preeclampsia and gestational hypertension [4]. In the present study, maternal smoking was considered a possible mediator of socioeconomic status. We primarily aimed to understand the association between SHS and HDP risk; thus, we adjusted for socioeconomic factors, including educational levels, income, and maternal smoking, as confounders for SHS.

Several studies have indicated an association between SHS exposure and increased hypertension risk in the nonpregnant population. For example, Yang et al. conducted a large cross-sectional study using data from over 5 million women and their husbands from the National Free Prepregnancy Checkup Projects in China. The authors observed that having husbands who smoked were significantly associated with an increased prevalence of hypertension among their wives in categorical, dose‒response, and cumulative manners [24]. Additionally, Makris et al. reported that masked hypertension (defined as the mean clinic systolic and diastolic BP of <140 and 90 mmHg, respectively, conjointly with daytime systolic or diastolic BP of >135 and 85 mmHg, respectively) was associated with SHS exposure in a dose-related manner [25]. Seki et al. reported a relationship between SHS exposure and increased BP at home in Japanese nonpregnant women; compared to that in nonpregnant women without SHS exposure, home BP measurements differed by ~3–4 mmHg in nonpregnant women exposed to SHS at home and at the workplace [26].

The present study is also the first to evaluate the PAF of SHS exposure to HDP during pregnancy. The combined estimate of PAF due to SHS was 3.8%, i.e., approximately twice 1.8% of PAF due to perinatal smoking among pregnant women; the impact of SHS on HDP is larger than that of smoking in pregnant women; therefore, it is important to prevent SHS for better health of pregnant women. The PAF of SHS exposure to cancer has been investigated in Australia [27], Spain [28], Korea [29], and Japan [30]. Each of these studies concluded that SHS exposure was a preventable risk factor for cancer incidence. In a perinatal epidemiologic study, Ojima et al. estimated PAFs of 15.6, 1.1, and 7.0 for SHS exposure, at home and at the workplace, and active smoking, respectively, to low birth weights [31]. These studies also showed that public education on the avoidance of SHS exposure is very important.

Despite relatively comparable adjusted odds ratios, we observed a higher PAF of SHS exposure to HDP than that for maternal smoking to HDP. The number of women exposed to SHS perinatally and who smoked in early pregnancy were 37,546 (49.4%) and 13,226 (17.4%), respectively. The difference between the PAF of SHS exposure to HDP and that of maternal smoking to HDP likely derives from the difference in the population exposed. The avoidance of SHS exposure has a higher impact on public health than individual smoking discontinuation.

The present study has several limitations. First, we could not classify HDP into gestational hypertension, preeclampsia, and other specific conditions due to the nature of the questionnaire employed in this study. Therefore, we could not assess the effect of SHS exposure on each subtype of HDP. Furthermore, preeclampsia is less common among smokers (smoking-preeclampsia paradox) [32]; however, we could not assess the influence of the smoking-preeclampsia paradox on SHS exposure. Second, in the present study, PAFs could not reveal the population of pregnant women in Japan because many participants were excluded due to insufficient information on baseline characteristics or complications. Subsequently, we performed the same analysis, including participants with miscarriages, abortions, or complications, and reclassified the participants with missing information into another category. Although we detected similar trends in another category, the PAFs were larger than those of the analysis performed after exclusions. Third, although we adjusted for possible confounding factors available in the datasets, unmeasured confounding factors could also affect the risk of HDP development. For example, family history of hypertension, diabetes mellitus, and HDP are important factors that influence the development of HDP; however, these were not considered. Fourth, the analysis was conducted using the questionnaire item of SHS exposure before pregnancy as an indicator of SHS up to early pregnancy. There should be careful application of our study results because the decision to avoid SHS exposure may change with the cognition of pregnancy. Furthermore, information related to SHS exposure before pregnancy was collected using self-report questionnaires, which could cause underreporting of SHS exposure and its duration, leading to misclassification. Additionally, several previous studies reported high concordance rates between self-administered questionnaires and biomarkers [33, 34], suggesting some consistency with the present results. Finally, we could not examine the genetic background of the participants.

From the Asian perspective, the effects of smoking on the population may be influenced by differences in genetic background, as discussed in previous meta-analyses [11]. The risk of hypertension is higher in people with slow nicotine metabolism than in others [35], and the allele frequency of slow nicotine metabolism was high in the Chinese and Japanese populations, at 15% and 20%, respectively, whereas it was <5% in Caucasians [36]. These genetic background differences should be taken into account when considering strategies against SHS exposure.

In conclusion, women with greater SHS exposure showed a higher risk of HDP after adjustment for possible confounding factors. Thus, relevant strategies are needed to reduce SHS exposure in the population.

## Supplementary information

Supplementary Materials
