# Supplementary material for: Secondhand smoke exposure is associated with the risk of hypertensive disorders of pregnancy: the Japan Environment and Children’s Study
Source: Hypertens Res. 2023 Feb 3;46(4):834–44. doi: 10.1038/s41440-022-01144-3 (PMC10073017; doi:10.1038/s41440-022-01144-3)
Supplement: Supplementary file 1 — Supplementary Materials [file 41440_2022_1144_MOESM1_ESM.pdf]

Supplemental Table 1. Basic characteristics (total n=75,826)

		Frequency of SHS exposure before pregnancy		
		rarely	1-3 days/week	4-7 days/week
		(n = 38,368)	(n = 17,819)	(n = 19,639)
Duration of SHS exposure (/day)(%)				
	<1 hour	99.83	81.48	60.29
	1-2 hours	0.11	13.47	20.75
	≥2 hours	0.05	5.05	18.95
Participant's background				
Age (years)				
	mean (SD)	32.18 (4.57)	31.03 (4.86)	30.04 (5.29)
	<20	0.17	0.42	1.45
	20 - 24	4.48	8.62	14.35
	25 - 29	24.27	30.38	31.50
	30 - 34	39.26	35.24	31.06
	35 - 39	26.51	21.25	17.95
	≥40	5.31	4.10	3.70
Primipara (%)		37.31	46.17	48.33
BMI before pregnancy (kg/m^2) (%)				
	mean (SD)	21.04 (3.03)	21.29 (3.29)	21.43 (3.55)
	<18.5	15.98	15.70	16.17
	18.5 - 24.9	75.12	73.46	71.29
	≥25.0	8.90	10.85	12.54
Weight gain during pregnancy (kg) (SD) (n=74,275)		9.95 (5.80)	10.27 (3.94)	11.01 (4.38)
Smoking before pregnancy (%)				
	no smoking	70.62	59.40	35.97
	quit before pregnancy	23.49	26.10	21.46
	quit during pregnancy	5.06	11.69	29.47
	continued smoking	0.82	2.81	13.09
Alcohol consumption at entry (%)				
	no drinking	37.46	32.34	30.21
	quit before or during pregnancy	52.09	56.88	60.27
	continued drinking	10.45	10.78	9.52
K6 score ≥ 13 (%)		2.43	3.30	5.03
Educational level (%)				
	junior high school	1.74	3.77	9.63
	high school	23.05	30.85	44.25
	college	75.21	65.39	46.11
Family income (JPY) (%)				
	$<200 \times 10^4$	3.02	5.34	9.61
	$200 - 399 \times 10^4$	29.58	35.14	42.77
	$400 - 599 \times 10^4$	35.58	33.15	29.21
	$600 - 799 \times 10^4$	18.79	15.66	11.21
	$\geq 800 \times 10^4$	13.03	10.71	7.20
Sodium intake (mg/day)	median (inter quartile range)	2864 (2157-3784)	2884 (2132-3881)	2872 (2082-3932)
Pottasium intake (mg/day)	median (inter quartile range)	2137 (1617-2832)	2104 (1560-2823)	2029 (1477-2770)
Calcium intake (mg/day)	median (inter quartile range)	468 (327-655)	450 (310-644)	420 (280-616)
Magnecium intake (mg/day)	median (inter quartile range)	223 (173-290)	221 (169-290)	215 (160-285)
Salt intake (g/day)	median (inter quartile range)	7.2 (5.4-9.5)	7.3 (5.4-9.8)	7.2 (5.2-9.9)
Total Energy intake (KJ/day)	median (inter quartile range)	7064 (5803-8718)	7166 (5778-8883)	7103 (5648-9002)
History of HDP (%)		1.22	1.3	1.36
History of GDM (%)		0.66	0.59	0.65
Patner's Background				
Partner's smoking before pregnancy (%)				
	no smoking	37.65	25.20	10.23
	quit before pregnancy	31.23	22.65	9.81
	quit during pregnancy	1.94	2.96	3.29
	continued smoking	29.18	49.19	76.67
Partner's educational level (%)				
	junior high school	3.22	6.45	14.71
	high school	30.43	37.41	46.31
	college	66.34	56.14	38.98
Outcome				
Prevalence of HDP (%)		2.64	3.01	3.38

SD: Standard deviation
BMI: Body mass index
SHS: secondhand smoke
JPY: Japanese yen
HDP: Hypertensive disorders of pregnancy
K6: Kessler psychological distress scale

Supplement Table 2. Results of the univariate analyses on HDP for the covariates (total n =75,826)

		RR	95% CI
Frequency of SHS exposure	rarely	Reference	
	1–3 days/week	1.141	1.030 – 1.265
	4–7 days/week	1.281	1.163 – 1.410
Duration of SHS exposure	< 1 hour	Reference	
	1–2 hours	1.202	1.048 – 1.380
	≥2 hours	1.374	1.182 – 1.597
Age (years)	<20	Reference	
	20–24	0.775	0.443 – 1.355
	25–29	0.786	0.457 – 1.351
	30–34	0.841	0.490 – 1.443
	35–39	1.196	0.696 – 2.053
	≥40	1.812	1.043 – 3.147
Parity	primipara	1.952	1.796 – 2.121
	multipara	Reference	
BMI (kg/m^2)	<18.5	0.699	0.606 – 0.806
	18.5 – 24.9	Reference	
	≥25.0	2.967	2.702 – 3.257
Smoking before pregnancy	no smoking	Reference	
	quit before pregnancy	1.050	0.950 – 1.161
	quit during pregnancy	1.147	1.016 – 1.295
	continued smoking	1.210	1.002 – 1.460
Alcohol consumption	no drinking	Reference	
	quit before or during pregnancy	0.947	0.867 – 1.034
	continued drinking	0.884	0.761 – 1.027
K6 score	<13	Reference	
	≥13	1.082	0.868 – 1.350
Maternal educational level	junior high school	1.088	0.889 – 1.331
	high school	1.143	1.047 – 1.248
	college	Reference	
Family income (× 10^4 JPY)	<200	Reference	
	200 – 399	0.902	0.781 – 1.041
	400 – 599	0.870	0.753 – 1.005
	600 – 799	0.923	0.786 – 1.084
	≥800	0.821	0.821 – 1.011
Sodium intake	Q1	Reference	
	Q2	0.945	0.831 – 1.075
	Q3	0.914	0.803 – 1.041
	Q4	0.914	0.803 – 1.041
	Q5	1.018	0.897 – 1.155
Pottasium intake (mg/day)	Q1	Reference	
	Q2	0.925	0.813 – 1.052
	Q3	1.001	0.883 – 1.136
	Q4	0.861	0.755 – 0.981
	Q5	0.947	0.833 – 1.076
Calcium intake (mg/day)	Q1	Reference	
	Q2	1.086	0.958 – 1.230
	Q3	0.955	0.840 – 1.086
	Q4	0.917	0.805 – 1.044
	Q5	0.858	0.751 – 0.979
Magnecium intake (mg/day)	Q1	Reference	
	Q2	0.933	0.820 – 1.061
	Q3	0.985	0.867 – 1.118
	Q4	0.910	0.799 – 1.037
	Q5	0.956	0.841 – 1.087
Salt intake (g/day)	Q1	Reference	
	Q2	0.933	0.819 – 1.062
	Q3	0.941	0.827 – 1.071
	Q4	0.920	0.808 – 1.048
	Q5	1.007	0.886 – 1.144
Total Energy intake (KJ/day)	Q1	Reference	
	Q2	0.934	0.818 – 1.067
	Q3	1.000	0.878 – 1.139
	Q4	1.009	0.886 – 1.149
	Q5	1.089	0.958 – 1.237
History of gestational diabetes		1.410	0.916 – 2.170
History of HDP		6.678	5.804 – 7.685
Partner smoking status	no smoking	Reference	
	quit before pregnancy	0.971	0.865 – 1.090
	quit during pregnancy	0.784	0.580 – 1.061
	continued smoking	1.022	0.926 – 1.127
Partner's educational level	junior high school	1.002	0.847 – 1.187
	high school	1.138	1.044 – 1.240
	college	Reference	

RR: relative risk
CI: confidence interval
HDP: Hypertensive disorders of pregnancy
JPY: Japanese yen
K6: Kessler psychological distress scale
SHS: secondhand smoke
BMI: body mass index

Supplemental Table 3. Association between the frequency and duration of secondhand smoke exposure and HDP among whole participants

		Relative Risk	95% CI		
	Frequency of maternal SHS exposure before pregnancy				
Crude	rarely	Reference			
	1–3 days/week	1.141	1.030	–	1.265
	4–7 days/week	1.281	1.163	–	1.410
Model 1	rarely	Reference			
	1–3 days/week	1.062	0.958	–	1.179
	4–7 days/week	1.128	1.012	–	1.258
Model 2	rarely	Reference			
	1–3 days/week	1.042	0.939	–	1.156
	4–7 days/week	1.105	0.992	–	1.232
Model 3	rarely	Reference			
	1–3 days/week	1.039	0.937	–	1.153
	4–7 days/week	1.104	0.990	–	1.230
	Duration of maternal SHS exposure before pregnancy				
Crude	< 1 hour	Reference			
	1–2 hours	1.202	1.048	–	1.380
	≥2 hours	1.374	1.182	–	1.597
Model 1	< 1 hour	Reference			
	1–2 hours	1.053	0.914	–	1.213
	≥2 hours	1.212	1.034	–	1.422
Model 2	< 1 hour	Reference			
	1–2 hours	1.039	0.903	–	1.195
	≥3 hours	1.174	1.001	–	1.378
Model 3	< 1 hour	Reference			
	1–2 hours	1.038	0.903	–	1.194
	≥2 hours	1.178	1.003	–	1.382

Model 1: Adjusted for age, parity, body mass index before pregnancy, family income, maternal educational level, smoking status,Na, K, Ca, Mg and total energy intake, drinking status, and K6 score.

Model 2: Adjusted for a history of GDM and HDP in addition to the factors in Model

Model 3: Adjusted for partner's educational level in addition to the factors in Model

SHS: secondhand smoke

HDP: hypertensive disorders of pregnancy

GDM: gestational diabetes mellitus

K6: Kessler psychological distress scale

CI: confidence interval

Supplemental Table 4. PAFs of SHS exposure (stratified analysis)

	n	HDP	aRR	95% CI		
SHS exposure in non-smokers	44747	1256				
rarely	27097	698	Reference			
1-3 days/week	10585	313	1.046	0.917	-	1.195
4-7 days/week	7065	245	1.177	1.017	-	1.363
SHS exposure in smokers quitting smoking before pregnancy	17880	527				
rarely	9014	244	Reference			
1-3 days/week	4651	137	1.040	0.846	-	1.277
4-7 days/week	4215	146	1.170	0.908	-	1.372
SHS exposure in smokers quitting smoking during pregnancy	9813	316				
rarely	1942	65	Reference			
1-3 days/week	2083	71	0.884	0.638	-	1.224
4-7 days/week	5788	180	0.835	0.633	-	1.102
SHS exposure in smokers continuing smoking	3386	115				
rarely	315	6	Reference			
1-3 days/week	500	16	1.835	0.743	-	4.530
4-7 days/week	2571	93	1.799	0.806	-	4.018

† Adjusted for age, parity, body mass index before pregnancy, family income, educational levels of the participant and partner, drinking status of the participant, a history of HDP and GDM, and K6 score.

[a] Adjusted for smoking status of the participant in additional to †.

[b] Adjusted for SHS exposure (frequency) in additional to †.

[c] Composite PAF of each category.

PAF: population attributable fraction
HDP: hypertensive disorders of pregnancy
SHS: secondhand smoke
GDM: gestational diabetes mellitus
K6: Kessler psychological distress scale
aRR: adjusted relative risk
CI: confidence interval
